# Supplementary material for: Effectiveness of virtual reality-based rehabilitation on the upper extremity motor function of stroke patients: A protocol for systematic review and meta-analysis
Source: PLoS One. 2024 Nov 7;19(11):e0313296. doi: 10.1371/journal.pone.0313296 (PMC11542779; doi:10.1371/journal.pone.0313296)
Supplement: Attachment — Submitted filename: PONE-D-24-16134.pdf [file pone.0313296.s002.pdf]

PLOS ONE

Efficacy of virtual reality-based rehabilitation on the upper extremity deficits of stroke patients: A protocol for systematic review and meta-analysis

--Manuscript Draft--

**Manuscript Number:**	PONE-D-24-16134
**Article Type:**	Study Protocol
**Full Title:**	Efficacy of virtual reality-based rehabilitation on the upper extremity deficits of stroke patients: A protocol for systematic review and meta-analysis
**Short Title:**	Efficacy of virtual reality-based rehabilitation on the upper extremity deficits of stroke patients
**Corresponding Author:**	Jiali Zhang<br>Kunming Municipal Hospital of Traditional Chinese Medicine<br>Kunming, CHINA
**Keywords:**	stroke; upper extremity deficits; virtual reality; protocol; meta-analysis; systematic review
**Abstract:**	Introduction<br>Upper extremity deficits (UED) is a common and impactful complication among stroke survivors. Virtual reality (VR)-based rehabilitation holds potential for enhancing rehabilitation intensity and engagement by stimulating tasks. While several clinical studies have examined the efficacy and safety of VR-based rehabilitation, there is a need for further research to improve consistency in outcomes.<br>Methods<br>The study will incorporate randomized controlled trials (RCTs) concerning the effects of VR-based rehabilitation on upper extremity (UE) function in stroke survivors. A comprehensive search of databases including PubMed, Embase, Cochrane Library, Web of Science, Scopus, Cinahl, China National Knowledge Infrastructure (CNKI), Wan-fang, and Chinese Biology Medicine Database will be performed from inception to the start of the study. Primary outcomes will focus on upper limb motor function assessments such as the Fugl-Meyer Upper Extremity (FMUE), Box and Block Test (BBT), Wolf Motor Function Test (WMFT), and Action Research Arm Test (ARAT). Secondary outcomes related to activities of daily living will include the Barthel Index (BI) and Functional Independence Measure (FIM). Research selection, data extraction, and quality assessment will be independently conducted by two researchers. The recently revised Cochrane risk of bias tool will be employed to evaluate study quality. Meta-regression and subgroup analyses will be utilized to identify effective therapy delivery modes and patterns. The assessment, development, and evaluation of recommendations approach will be applied to achieve a robust conclusion.<br>Conclusion<br>This study provides a rigorous synthesis to assess the optimal parameters of VR-based rehabilitation for enhancing UE function in stroke survivors. A precise, transparent and standardized review process is anticipated to yield evidence-based recommendations for integrating VR technology into the treatment of UED in stroke patients. This will offer clinicians valuable insights into rehabilitation strategies.
**Order of Authors:**	Jiali Zhang
	Jie Yang
	Qiuzhu Xu
	Yan Xiao
	Liang Zuo
	Enli Cai
**Additional Information:**	
**Question**	**Response**
**Financial Disclosure**	The author(s) received no specific funding for this work.

Powered by Editorial Manager® and ProduXion Manager® from Aries Systems Corporation

Enter a financial disclosure statement that describes the sources of funding for the work included in this submission. Review the submission guidelines for detailed requirements. View published research articles from *PLOS ONE* for specific examples.<br><br>This statement is required for submission and **will appear in the published article** if the submission is accepted. Please make sure it is accurate.<br><br>**Funded studies**<br>Enter a statement with the following details:<br>• Initials of the authors who received each award<br>• Grant numbers awarded to each author<br>• The full name of each funder<br>• URL of each funder website<br>• Did the sponsors or funders play any role in the study design, data collection and analysis, decision to publish, or preparation of the manuscript?<br><br>Did you receive funding for this work?	
**Competing Interests**<br><br>Use the instructions below to enter a competing interest statement for this submission. On behalf of all authors, disclose any competing interests that could be perceived to bias this work—acknowledging all financial support and any other relevant financial or non-financial competing interests.<br><br>This statement is required for submission and **will appear in the published article** if the submission is accepted. Please make sure it is accurate and that any funding sources listed in your Funding Information later in the submission form are also declared in your Financial Disclosure statement.	The authors have declared that no competing interests exist.

View published research articles from *PLOS ONE* for specific examples.<br><br>**NO authors have competing interests**<br><br>Enter: *The authors have declared that no competing interests exist.*<br><br>**Authors with competing interests**<br><br>Enter competing interest details beginning with this statement:<br><br>*I have read the journal's policy and the authors of this manuscript have the following competing interests: [insert competing interests here]*<br><br>* typeset	
**Ethics Statement**<br><br>Enter an ethics statement for this submission. This statement is required if the study involved:<br><br>• Human participants<br>• Human specimens or tissue<br>• Vertebrate animals or cephalopods<br>• Vertebrate embryos or tissues<br>• Field research<br><br>Write "N/A" if the submission does not require an ethics statement.<br><br>General guidance is provided below. Consult the submission guidelines for detailed instructions. **Make sure that all information entered here is included in the Methods section of the manuscript.**	N/A

Powered by Editorial Manager® and ProduXion Manager® from Aries Systems Corporation

**Format for specific study types**<br><br>**Human Subject Research (involving human participants and/or tissue)**<br>• Give the name of the institutional review board or ethics committee that approved the study<br>• Include the approval number and/or a statement indicating approval of this research<br>• Indicate the form of consent obtained (written/oral) or the reason that consent was not obtained (e.g. the data were analyzed anonymously)<br><br>**Animal Research (involving vertebrate animals, embryos or tissues)**<br>• Provide the name of the Institutional Animal Care and Use Committee (IACUC) or other relevant ethics board that reviewed the study protocol, and indicate whether they approved this research or granted a formal waiver of ethical approval<br>• Include an approval number if one was obtained<br>• If the study involved *non-human primates*, add *additional details* about animal welfare and steps taken to ameliorate suffering<br>• If anesthesia, euthanasia, or any kind of animal sacrifice is part of the study, include briefly which substances and/or methods were applied<br><br>**Field Research**<br><br>Include the following details if this study involves the collection of plant, animal, or other materials from a natural setting:<br>• Field permit number<br>• Name of the institution or relevant body that granted permission	
**Data Availability**<br><br>Authors are required to make the data underlying their research findings fully available, without restriction.<br><br>If the manuscript reports pilot or preliminary results, the data underlying those results must be made available at the time of publication. A Data Availability	Yes - Results are reported and the data are fully available

Statement describing where the data can be found is required at submission. Your answers to this question constitute the Data Availability Statement and will be published in the article, if accepted. When legal or ethical requirements prohibit public data sharing, authors must indicate how researchers may obtain access to the data. See the PLOS Data Policy and FAQ for detailed information.<br><br>**Important:** Stating 'data available on request from the author' is not sufficient. If your data are only available upon request, select 'No' for the first question and explain your situation in the text box.<br><br>For protocols without pilot or preliminary data, authors are strongly encouraged to state how they plan to share research data from their study when it is completed or published.<br><br>If your study includes pilot data, will they be made fully available without restriction?	
**Describe where the data may be found in full sentences. If you are copying our sample text, replace any instances of XXX with the appropriate details.**<br><br>• If **no pilot data are reported** in the manuscript, please enter the following: *No datasets were generated or analysed during the current study. All relevant data from this study will be made available upon study completion.*<br>• If the manuscript reports pilot data **held in a public repository**, include URLs, accession numbers or DOIs. If this information will only be available after acceptance, indicate this by ticking the box below. For example: *All XXX files are available from the XXX database (accession number(s) XXX, XXX.).*<br>• If the manuscript reports pilot data contained **within the manuscript and/or**	Deidentified research data will be made publicly available when the study is completed and published.

**Supporting Information files**, enter the following:<br>*All relevant data are within the manuscript and its Supporting Information files.*<br>• If neither of these applies but you are able to provide **details of access elsewhere**, with or without limitations, please do so. For example:<br><br>*Data cannot be shared publicly because of [XXX]. Data are available from the XXX Institutional Data Access / Ethics Committee (contact via XXX) for researchers who meet the criteria for access to confidential data.*<br><br>*The data underlying the results presented in the study are available from (include the name of the third party and contact information or URL).*<br>• This text is appropriate if the data are owned by a third party and authors do not have permission to share the data.<br><br>For study protocols with data management or sharing plans, authors are encouraged to briefly describe in their Data Availability Statement how data they generate will be made accessible when the study is completed. For example:<br>• Deidentified research data will be made publicly available when the study is completed and published.<br><br>* typeset	
Additional data availability information:	

Powered by Editorial Manager® and ProduXion Manager® from Aries Systems Corporation

Manuscript Click here to access/download;Manuscript;Manuscript.docx

Efficacy of virtual reality-based rehabilitation on the upper extremity deficits of stroke patients: A protocol for systematic review and meta-analysis

Jiali Zhang[1], Jie Yang[1*], Qiuzhu Xu[2], Yan Xiao[1], Liang Zuo[3], Enli Cai[4*]

[1]Kunming Municipal Hospital of Traditional Chinese Medicine, Kunming, Yunnan Province, China,

[2]Haikou Orthopedic and Diabetes Hospital of Shanghai Sixth People's Hospital, Haikou, Hainan Province, China, [c]Kunming

[3]The Second Affiliated Hospital of Yunnan University of Traditional Chinese Medicine, Yunnan Province, China

[4]Yunnan University of Traditional Chinese Medicine, Kunming, Yunnan Province, China

*y15925112913@163.com(EC);*kmszcssd@163.com(JY)

Funding

The authors have not declared a specific grant for this research from any funding agency in the public, commercial or not-for-profit sectors.

Competing interests

The authors have no conflicts of interest to disclose.

Data availability

Data sharing not applicable to this article as no datasets were generated or analyzed during the current study.

Abstract

Introduction

Upper extremity deficits (UED) is a common and impactful complication among stroke survivors. Virtual reality (VR)-based rehabilitation holds potential for enhancing rehabilitation intensity and engagement by stimulating tasks. While several clinical studies have examined the efficacy and safety of VR-based rehabilitation, there is a need for further research to improve consistency in outcomes.

Methods

The study will incorporate randomized controlled trials (RCTs) concerning the effects of VR-based rehabilitation on upper extremity (UE) function in stroke survivors. A comprehensive search of databases including PubMed, Embase, Cochrane Library, Web of Science, Scopus, Cinahl, China National Knowledge Infrastructure (CNKI), Wan-fang, and Chinese Biology Medicine Database will be performed from inception to the start of the study. Primary outcomes will focus on upper limb motor function assessments such as the Fugl-Meyer Upper Extremity (FMUE), Box and Block Test (BBT), Wolf Motor Function Test (WMFT), and Action Research Arm Test (ARAT). Secondary outcomes related to activities of daily living will include the Barthel Index (BI) and Functional Independence Measure (FIM). Research selection, data extraction, and quality assessment will be independently conducted by two researchers. The recently revised Cochrane risk of bias tool will be employed to evaluate study quality. Meta-regression and subgroup analyses will be utilized to identify effective therapy delivery modes and patterns. The assessment, development, and evaluation of recommendations approach will be applied to achieve a robust conclusion.

Conclusion

This study provides a rigorous synthesis to assess the optimal parameters of VR-based rehabilitation for enhancing UE function in stroke survivors. A precise, transparent and standardized review process is anticipated to yield evidence-based recommendations for integrating VR technology into the treatment of UED in stroke patients. This will offer clinicians valuable insights into rehabilitation strategies.

Introduction

Stroke is a leading cause of disability worldwide, affecting almost 14 million people annually [1, 2]. While stroke mortality rates show a declining trend, the number of individuals experiencing the consequences of stroke is increasing due to population growth and ageing [3]. This upward trend has led to substantial, enduring disabilities in adults [4].

Approximately 75% of stroke survivors will demonstrate enduring deficits in motor control of their arm and hand, leading to enormous personal and societal consequences [5]. This increase in the number of stroke survivors highlights the growing need for rehabilitation services [6]. A common and severe disabling complication of stroke is UED [7]. These deficits persist partly due to the failure of current nonrepresentational approaches to substantially reduce upper-limb impairment [8]. Common manifestations of UED include loss of strength, reduced flexibility, abnormal cooperative interaction incursion, and muscle tension disorders [9]. These impairments can cause disabilities in common activities such as reaching, picking up objects, and holding onto objects [10].

Moreover, the restoration of UE function is a complex process with poor prognoses [11], which significantly affects patients' independence in daily activities and greatly reduces their quality of life. This places a considerable burden on both families and society. Consequently, it is crucial to enhance

the functional capacity of the UE and promote greater levels of independence in individuals after a stroke. Contemporary clinical strategies for UE rehabilitation rely on fostering neuroplasticity post-brain impairments [12]. Intensive and extensive task-specific training emphasizing numerous repetitions, has emerged as crucial in motor therapy following a stroke [13].

According to the guideline, postural training and task-oriented upper limb training have the potential to positively influence on upper limb motor control [14]. Currently, the field of neurorehabilitation encompasses several technologies that hold promise for addressing various neurological dysfunctions [15]. Among these, VR stands out as an innovative intervention in rehabilitation nursing, offering an enriched environment conducive to task-specific training and delivering multimodal feedback to promote functional recovery [16]. VR interventions for motor impairments showed positive rehabilitative effects in stroke survivors [17]. The three fundamental concepts of VR are immersion, imagination, and interaction [18]. Patients can immerse themselves in simulated scenarios, interact with their environment by engaging imagery, and receive real-time feedback, fostering immersive experiences conducive to motor rehabilitation. In parallel with usual rehabilitation therapy programs, VR not only supplements existing strategies but also motivates patients to engage in more purposeful practices, thereby intensifying the efficacy of targeted movements.

However, there is still no consensus in the field of UE rehabilitation regarding the specific types, duration, and intensity of VR training required to assess its clinical efficacy [19]. Moreover, a previous meta-analysis indicated a significant increase in the utilization of VR-based training for UE rehabilitation, resulting in varied outcomes. Therefore, it is necessary to implement a comprehensive and standardized protocol for conducting a systematic review and meta-analysis to evaluate the

efficacy of VR training in ameliorating UED after a stroke. Furthermore, favorable outcomes from this study may prompt further investigation into the optimal dosage of VR training to enhance upper limb function in stroke survivors, thereby advancing stroke clinical practice and informing the development of future clinical practice guidelines in the future.

Methods and analysis

For the design and reporting of this systematic review, we will strictly follow the Preferred Reporting Items for Systematic Reviews and Meta-Analysis Protocols (PRISMA-P) 2015 [20] and AMSTAR2 [21]. The PRISMA-P checklist is shown in S1 Table.

Criteria for study selection

In general, studies will be screened and selected based on PICOS format as follows:

Types of study. Only studies with the design of RCTs with at least two groups in which one of them undergoing VR-based training plus conventional therapy, while the control group receives only conventional therapy, will be eligible for inclusion. Studies evaluating any degree of intensity and different duration within the realm of VR will be incorporated. Additionally, studies employing both immersive and non-immersive VR modalities, as well as those utilizing commercially available gaming consoles, will be eligible for inclusion.

Types of participants. Patients diagnosed with stroke will be considered regardless of their age, sex, severity, or disease duration [22].

Types of intervention. The experimental group (EG) will be subjected to VR-based training plus conventional therapy, whereas the control group (CG) will undergo treatment involving conventional training, occupational therapy, physical therapy, usual care alone, or conventional rehabilitation, encompassing any therapeutic activities targeting impairment, activity, or participation

levels.

Types of outcome measure. The primary outcomes focused on activity and activity limitations [23]. This included indicators of the ability to perform an UE function (using the arm and hand) and could include tools such as: (1) FMUE [24]; (2) BBT [25]; (3) WMFT [26]; (4) ARAT [27]. Secondary outcomes focused on activities of daily living, such as BI [28] and FIM [29]. Alternatively, additional outcome measures of interest pertaining to the restoration of upper limb functionality following a stroke will be considered.

Data sources and search strategy

PubMed, Embase, Cochrane Library, Web of Science, Scopus, Cinahl, CNKI, Wan-fang, and Chinese Biology Medicine Database will be comprehensively searched from inception to the start of the study. Medical subject heading (MeSH) terms related and text words will be adopted, mainly including stroke, cerebrovascular disorders, virtual reality, virtual reality exposure therapy, upper extremity, upper limb, arm. Grey literature such as theses and articles located via the snowball, dissertations and conference proceedings, will also be consulted.

Data screening and extraction

Two independent authors conducted an initial assessment of studies for potential incorporation. The screening process encompassed the scrutiny of titles and abstracts pinpointed during the search, succeeded by a comprehensive analysis of the complete texts against the predetermined inclusion standards. Excluded studies after full-text appraisal will be meticulously recorded and elucidated concerning the rationale for their exclusion. Any discrepancies in data extraction were resolved through consensus or by consulting a third author. The research flowchart is presented in Fig 1.

Data extraction will be performed with a pre-piloted, standardized form. The following

information will extracted: journal title, first author, publication year, country, patient demographics (stroke recovery stage, stroke duration, sample size, sex, mean age), control and experimental parameters (VR session duration, VR training frequency, VR period), outcomes, duration of intervention, among others. Any discrepancies will be resolved through consultation with a third author.

Data synthesis and analysis

Data synthesis will be conducted utilizing STATA (version 17.0, StataCorp LLC). The outcomes included in the analysis comprised continuous data. Pooled results were estimated by calculating the mean difference (MD) or standardized mean difference (SMD), along with 95% confidence intervals (CI) [30]. The *p*-values were two sided, with an alpha level of 0.05 considered significant [31].

Assessment of risk of bias

Two reviewers will independently evaluate the risk of bias using the RoB 2 tool (revised tool for risk of bias in randomized studies) [32]. This tool encompasses seven domains: random sequence generation, allocation concealment, blinding of participants and personnel, blinding of outcome assessment, incomplete outcome data, selective reporting, and other biases. The risk of bias will be categorized as "low", "unclear", or "high" for each domain. These seven domains will be appraised independently by two reviewers, and discrepancies will be addressed by consulting a third reviewer.

Assessment of heterogeneity

In evaluating heterogeneity between trials, the I^2 statistic was employed. The methodology entailed the following procedures: if the I^2 test was <50%, the fixed effects model was applied for data synthesis. Conversely, the random-effects model was applied for data synthesis if the I^2 test was between 50% and 75% [33]. If the I^2 test is >75%, meta-regression analyses and subgroup analysis

would be conducted to identify possible reasons. A sensitivity analysis will be conducted to evaluate the robustness of the primary decisions made during the review process.

Assessment of evidence quality

For each aggregated or individual effect size (ES) of functioning, the certainty of the evidence body will be independently rated by two reviewers with GRADE [34]. Any inconsistencies will be resolved through consultation with a third reviewer. Summary of findings tables will be generated utilizing GRADEPro-GDT.

Information on all primary and secondary outcomes derived from our review will be incorporated. The quality of the evidence will be undergo evaluation based on five factors: 1) limitations in trial design and execution of available trials; 2) indirect evidence; 3) unexplained heterogeneity or inconsistency of results; 4) imprecision in effect estimates; and 5) potential publication bias.

If the number of studies exceeds ten, a funnel plot will be constructed and scrutinized to investigate potential biases in studies and publications in accordance with the guidelines outlined in the Cochrane Handbook for Systematic Reviews of Interventions [32]. Begg's and Egger's tests will be employed to evaluate the publication bias of these trials and generate the publication bias plot. If the funnel plots are found to be asymmetrical, we will try to interpret funnel plot asymmetry [35].

Ethical considerations

This review will not entail the utilization of private individual information nor infringe upon patient rights, thus obviating the necessity for ethical approval. The results of this review will be disseminated through academic publications and conference presentations.

Discussion

UE function deficits represent a prevalent challenge encountered by stroke survivors, significantly

associated with increased levels of physical impairment, disability, and reduced quality of life. Moreover, UE function impairment poses a significant impact on stroke patients, their families and the broader societal framework. The restoration of optimal UE function is crucial for reinstating independence in daily activities.

Over the past twenty years, there has been a notable increase in research on the effectiveness of VR in treating motor function. The use of VR training to enhance arm motor function after a stroke shows great promise. This method allows individuals to participate in engaging training sessions that involve multiple repetitions, prominent stimuli, and challenging tasks, all of which are believed to elicit adaptive neuroplastic changes [36]. The literature supports the notion that VR interventions may yield favorable outcomes on the impairment of UE, potentially outperforming conventional therapeutic approaches. Previous research [37] has illustrated that VR-based training technology has the potential to induce cortical reorganization of neuromotor pathways. Before VR training, activation occurred bilaterally in the patient's primary motor cortex, ipsilateral sensorimotor cortex, and motor accessory area cortex. Following the training, these regions were inhibited, while contralateral sensorimotor cortical areas were activated, thereby promoting compensation and performance of lost motor functions. The use of VR technology in rehabilitation settings allows patients to receive prompt feedback on their task execution, along with visual and auditory stimuli that often capture their interest. VR engenders motivation for active engagement in therapy sessions, as patients derive enjoyment from participating in tasks. Moreover, VR interventions can enhance stability in activities of daily living, thereby augmenting the independence of stroke patient [38].

Currently, the field of UE rehabilitation faces the challenge of customizing VR-based training to meet the distinct requirements of individuals. This poses a potential obstacle for clinicians who wish

to integrate VR into clinical practice. Therefore, developing a comprehensive feasibility protocol is essential to amalgamate evidence regarding various types, frequencies, duration, and intensities of VR-based rehabilitation, ensuring clinical efficacy in addressing UED among stroke survivors.

To the best of our knowledge, this is the first systematic review and meta-analysis to synthesize the efficacy of of VR-based rehabilitation on UED in stroke patients. This paper outlines a rigorous systematic review protocol to evaluate the efficacy and safety of VR in rehabilitating UE function. This proposed review will be conducted following the latest guidelines of the Cochrane Handbook for Systematic Reviews of Interventions. The statistical method of meta-regression will be used to investigate potential factors affecting VR outcomes. Subsequently, subgroup analyses will be conducted based on the findings. In addition, the outcome indicators formulated for evaluating upper limb function were meticulous and covered a wide array of outcome measures across the diverse RCTs included. We will initially extract dose-effect relationships concerning training volume to provide guidance to clinicians and practitioners in formulating effective VR-based training protocols for UE motor function. Further research is needed to reveal optimal dose-response relationships following VR training.

Furthermore, we will address various relevant considerations in the systematic review and meta-analysis process. While an exhaustive literature search will be conducted, it is acknowledged that not all relevant RCTs studies adhering to the protocol may be included. We will exercise caution in interpreting results, particularly in instances of limited study and patient inclusion, as well as in trials employing multiple treatment methodologies. Another anticipated limitation of this study includes potential heterogeneity arising from variations in stroke severity among participants in the incorporated studies, which may compromise result reliability.

Despite these potential limitations, our aspiration is for the study outcomes to serve as a valuable synthesis of the available evidence, offering preliminary insights for both current clinical practices addressing UED and guiding future research endeavors. We believe this protocol is promising and it will empower the healthcare professionals, caregivers and especially stroke patients with UED.

Supporting information

S1 Table. PRISMA-P (Preferred Reporting Items for Systematic review and Meta-Analysis Protocols) 2015 checklist: Recommended items to address in a systematic review protocol*.

(DOC)

Acknowledgment

The authors thank teacher Cheng Qing for consultation regarding the statistical analysis and study design.

Author contributions

Conceptualization: Enli Cai, Jiali Zhang.

Data curation: Qiuzhu Xu, Yan Xiao, Liang Zuo.

Formal analysis: Jiali Zhang, Jie Yang.

Investigation: Qiuzhu Xu, Yan Xiao.

Methodology: Jiali Zhang, Jie Yang.

Project administration: Jiali Zhang

Resources: Qiuzhu Xu, Yan Xiao.

Software: Jie Yang, Liang Zuo.

Supervision: Enli Cai.

Validation: Jiali Zhang, Jie Yang, Qiuzhu Xu, Yan Xiao, Liang Zuo.

Writing – original draft: Jiali Zhang, Jie Yang.

Writing – review & editing: Enli Cai, Jiali Zhang, Jie Yang.

References

1. Fan Y, Song Z, Zhang M. Emerging frontiers of artificial intelligence and machine learning in ischemic stroke: a comprehensive investigation of state-of-the-art methodologies, clinical applications, and unraveling challenges. EPMA J. 2023;14(4):645-61. Epub 2023/12/14. doi: 10.1007/s13167-023-00343-3. PubMed PMID: 38094579; PubMed Central PMCID: PMCPMC10713915.
2. Aguirre AO, Rogers JL, Reardon T, Shlobin NA, Ballatori AM, Brown NJ, et al. Stroke management and outcomes in low-income and lower-middle-income countries: a meta-analysis of 8535 patients. J Neurosurg. 2023;139(4):1042-51. Epub 2023/10/19. doi: 10.3171/2023.2.JNS222807. PubMed PMID: 37856884.
3. Ma Q, Li R, Wang L, Yin P, Wang Y, Yan C, et al. Temporal trend and attributable risk factors of stroke burden in China, 1990-2019: an analysis for the Global Burden of Disease Study 2019. Lancet Public Health. 2021;6(12):e897-e906. Epub 2021/11/29. doi: 10.1016/S2468-2667(21)00228-0. PubMed PMID: 34838196; PubMed Central PMCID: PMCPMC9047702.
4. Jianxiong Jiang YY. Small molecules targeting cyclooxygenase/prostanoid cascade in experimental brain ischemia: Do they translate? Med Res Rev. 2021;41(2):828-57. doi: 10.1002/med.21744. PubMed PMID: 33094540.
5. Virani SS, Alonso A, Aparicio HJ, Benjamin EJ, Bittencourt MS, Callaway CW, et al. Heart Disease and Stroke Statistics-2021 Update: A Report From the American Heart Association. Circulation. 2021;143(8):e254-e743. Epub 2021/01/28. doi: 10.1161/CIR.0000000000000950. PubMed PMID: 33501848.

6. Cathy M Stinear CEL, Steven Zeiler, Winston D Byblow. Advances and challenges in stroke rehabilitation. The Lancet Neurology. 2020;19(4):348-60. doi: 10.1016/s1474-4422(19)30415-6.
7. Muir KW, Majersik JJ. Connecting Upper Limb Functional Stroke Recovery to Global Disability Measures: Finding the Forest in the Trees. Neurology. 2021;96(14):643-4. Epub 2021/02/17. doi: 10.1212/WNL.0000000000011671. PubMed PMID: 33589536.
8. Powell MP, Verma N, Sorensen E, Carranza E, Boos A, Fields DP, et al. Epidural stimulation of the cervical spinal cord for post-stroke upper-limb paresis. Nat Med. 2023;29(3):689-99. Epub 2023/02/23. doi: 10.1038/s41591-022-02202-6. PubMed PMID: 36807682; PubMed Central PMCID: PMCPMC10291889.
9. Krakauer JW, Carmichael ST. Broken movement: the neurobiology of motor recovery after stroke: MIT Press; 2022.
10. Thieme H, Morkisch N, Mehrholz J, Pohl M, Behrens J, Borgetto B, et al. Mirror therapy for improving motor function after stroke. Cochrane Database Syst Rev. 2018;7(7):CD008449. Epub 2018/07/12. doi: 10.1002/14651858.CD008449.pub3. PubMed PMID: 29993119; PubMed Central PMCID: PMCPMC6513639.
11. Averta G, Barontini F, Catrambone V, Haddadin S, Handjaras G, Held JPO, et al. U-Limb: A multi-modal, multi-center database on arm motion control in healthy and post-stroke conditions. Gigascience. 2021;10(6). Epub 2021/06/19. doi: 10.1093/gigascience/giab043. PubMed PMID: 34143875; PubMed Central PMCID: PMCPMC8212873.
12. Coleman ER, Moudgal R, Lang K, Hyacinth HI, Awosika OO, Kissela BM, et al. Early Rehabilitation After Stroke: a Narrative Review. Curr Atheroscler Rep. 2017;19(12):59. Epub 2017/11/09. doi: 10.1007/s11883-017-0686-6. PubMed PMID: 29116473; PubMed Central PMCID: PMCPMC5802378.
13. JA K, TA J. Principles of experience-dependent neural plasticity: implications for rehabilitation after brain damage. J Speech Lang Hear Res. 2008;51(1):S225-39. doi: 10.1044/1092-4388(2008/018). PubMed PMID: 18230848.
14. Winstein CJ, Stein J, Arena R, Bates B, Cherney LR, Cramer SC, et al. Guidelines for Adult Stroke Rehabilitation and Recovery: A Guideline for Healthcare Professionals From the American Heart Association/American Stroke Association. Stroke. 2016;47(6):e98-e169. Epub 2016/05/06. doi: 10.1161/STR.0000000000000098. PubMed PMID: 27145936.

15. Hao J, Xie H, Harp K, Chen Z, Siu KC. Effects of Virtual Reality Intervention on Neural Plasticity in Stroke Rehabilitation: A Systematic Review. Arch Phys Med Rehabil. 2022;103(3):523-41. Epub 2021/08/06. doi: 10.1016/j.apmr.2021.06.024. PubMed PMID: 34352269.

16. Hsu HY, Kuo LC, Lin YC, Su FC, Yang TH, Lin CW. Effects of a Virtual Reality-Based Mirror Therapy Program on Improving Sensorimotor Function of Hands in Chronic Stroke Patients: A Randomized Controlled Trial. Neurorehabil Neural Repair. 2022;36(6):335-45. Epub 2022/03/29. doi: 10.1177/15459683221081430. PubMed PMID: 35341360.

17. Coscia M, Wessel MJ, Chaudary U, Millan JDR, Micera S, Guggisberg A, et al. Neurotechnology-aided interventions for upper limb motor rehabilitation in severe chronic stroke. Brain. 2019;142(8):2182-97. Epub 2019/07/02. doi: 10.1093/brain/awz181. PubMed PMID: 31257411; PubMed Central PMCID: PMCPMC6658861.

18. Le Du K, Septans AL, Maloisel F, Vanquaethem H, Schmitt A, Le Goff M, et al. A New Option for Pain Prevention Using a Therapeutic Virtual Reality Solution for Bone Marrow Biopsy (REVEH Trial): Open-Label, Randomized, Multicenter, Phase 3 Study. J Med Internet Res. 2023;25:e38619. Epub 2023/02/16. doi: 10.2196/38619. PubMed PMID: 36790852; PubMed Central PMCID: PMCPMC9978987.

19. Abbadessa G, Brigo F, Clerico M, De Mercanti S, Trojsi F, Tedeschi G, et al. Digital therapeutics in neurology. J Neurol. 2022;269(3):1209-24. Epub 2021/05/22. doi: 10.1007/s00415-021-10608-4. PubMed PMID: 34018047; PubMed Central PMCID: PMCPMC8136262.

20. Moher D, Shamseer L, Clarke M, Ghersi D, Liberati A, Petticrew M, et al. Preferred reporting items for systematic review and meta-analysis protocols (PRISMA-P) 2015 statement. 2015;4(1):1. doi: 10.1186/2046-4053-4-1. PubMed PMID: 25554246.

21. Shea BJ, Reeves BC, Wells G, Thuku M, Hamel C, Moran J, et al. AMSTAR 2: a critical appraisal tool for systematic reviews that include randomised or non-randomised studies of healthcare interventions, or both. BMJ. 2017;358:j4008. Epub 2017/09/25. doi: 10.1136/bmj.j4008. PubMed PMID: 28935701.

22. Report WS. Recommendations on stroke prevention, diagnosis, and therapy. Report of the WHO Task Force on Stroke and other Cerebrovascular Disorders. Stroke. 1989;20(10):1407-31.

23. Barclay RE, Stevenson TJ, Poluha W, Semenko B, Schubert J. Mental practice for treating upper extremity deficits in individuals with hemiparesis after stroke. Cochrane Database Syst Rev. 2020;5(5):CD005950. Epub 2020/05/26. doi: 10.1002/14651858.CD005950.pub5. PubMed PMID: 32449959; PubMed Central PMCID: PMCPMC7387111.

24. Fugl-Meyer AR, Jaasko L, Leyman I, Olsson S, Steglind S. The post-stroke hemiplegic patient. 1. a method for evaluation of physical performance. Scand J Rehabil Med. 1975;7(1):13-31. Epub 1975/01/01. PubMed PMID: 1135616.

25. Mathiowetz V, Volland G, Kashman N, Weber K. Adult norms for the Box and Block Test of manual dexterity. Am J Occup Ther. 1985;39(6):386-91. Epub 1985/06/01. doi: 10.5014/ajot.39.6.386. PubMed PMID: 3160243.

26. Wolf SL, Catlin PA, Ellis M, Archer AL, Morgan B, Piacentino A. Assessing Wolf motor function test as outcome measure for research in patients after stroke. Stroke. 2001;32(7):1635-9.

27. Hsieh CL, Hsueh IP, Chiang FM, Lin PH. Inter-rater reliability and validity of the action research arm test in stroke patients. Age Ageing. 1998;27(2):107-13. Epub 2005/11/22. doi: 10.1093/ageing/27.2.107. PubMed PMID: 16296669.

28. Mahoney FI, Barthel DW. Functional evaluation: the Barthel Index: a simple index of independence useful in scoring improvement in the rehabilitation of the chronically ill. Maryland state medical journal. 1965;77:1066–70.

29. Keitll R, Granger C, Hamilton B. The functional independence measure: a new tool for rehabilitstion. Adv Clin Rehabil. 1987;1:6-18.

30. Panagioti M, Panagopoulou E, Bower P, Lewith G, Kontopantelis E, Chew-Graham C, et al. Controlled Interventions to Reduce Burnout in Physicians: A Systematic Review and Meta-analysis. JAMA Intern Med. 2017;177(2):195-205. Epub 2016/12/06. doi: 10.1001/jamainternmed.2016.7674. PubMed PMID: 27918798.

31. Xie W, Wang Y, Xiao S, Qiu L, Yu Y, Zhang Z. Association of gestational diabetes mellitus with overall and type specific cardiovascular and cerebrovascular diseases: systematic review and meta-analysis. BMJ. 2022;378:e070244. Epub 2022/09/22. doi: 10.1136/bmj-2022-070244. PubMed PMID: 36130740.

32. Cumpston M, Li T, Page MJ, Chandler J, Welch VA, Higgins JP, et al. Updated guidance for trusted systematic reviews: a new edition of the Cochrane Handbook for Systematic Reviews of Interventions. Cochrane Database Syst Rev. 2019;10(10):ED000142. Epub 2019/10/24. doi: 10.1002/14651858.ED000142. PubMed PMID: 31643080; PubMed Central PMCID: PMCPMC10284251.

33. Wong SH, Gao Q, Tsoi KK, Wu WK, Tam LS, Lee N, et al. Effect of immunosuppressive therapy on interferon gamma release assay for latent tuberculosis screening in patients with autoimmune diseases: a systematic review and meta-analysis. Thorax. 2016;71(1):64-72. Epub 2015/12/15. doi: 10.1136/thoraxjnl-2015-207811. PubMed PMID: 26659461.

34. Yoshida M, Kinoshita Y, Watanabe M, Sugano K. JSGE Clinical Practice Guidelines 2014: standards, methods, and process of developing the guidelines. J Gastroenterol. 2015;50(1):4-10. Epub 2014/12/03. doi: 10.1007/s00535-014-1016-1. PubMed PMID: 25448314.

35. Smithers LG, Sawyer ACP, Chittleborough CR, Davies NM, Davey Smith G, Lynch JW. A systematic review and meta-analysis of effects of early life non-cognitive skills on academic, psychosocial, cognitive and health outcomes. Nat Hum Behav. 2018;2(11):867-80. Epub 2018/12/14. doi: 10.1038/s41562-018-0461-x. PubMed PMID: 30525112; PubMed Central PMCID: PMCPMC6277013.

36. Wilf M, Dupuis C, Nardo D, Huber D, Sander S, Al-Kaar J, et al. Virtual reality-based sensorimotor adaptation shapes subsequent spontaneous and naturalistic stimulus-driven brain activity. Cereb Cortex. 2023;33(9):5163-80. Epub 2022/10/27. doi: 10.1093/cercor/bhac407. PubMed PMID: 36288926; PubMed Central PMCID: PMCPMC10152055.

37. Bao X, Mao Y, Lin Q, Qiu Y, Chen S, Li L, et al. Mechanism of Kinect-based virtual reality training for motor functional recovery of upper limbs after subacute stroke. Neural Regen Res. 2013;8(31):2904-13. Epub 2014/09/11. doi: 10.3969/j.issn.1673-5374.2013.31.003. PubMed PMID: 25206611; PubMed Central PMCID: PMCPMC4146174.

38. Chen Y, Cao L, Xu Y, Zhu M, Guan B, Ming WK. Effectiveness of virtual reality in cardiac rehabilitation: A systematic review and meta-analysis of randomized controlled trials. Int J Nurs Stud. 2022;133:104323. Epub 2022/07/24. doi: 10.1016/j.ijnurstu.2022.104323. PubMed PMID: 35870329.

Figure Click here to access/download;Figure;Fig 1.tif

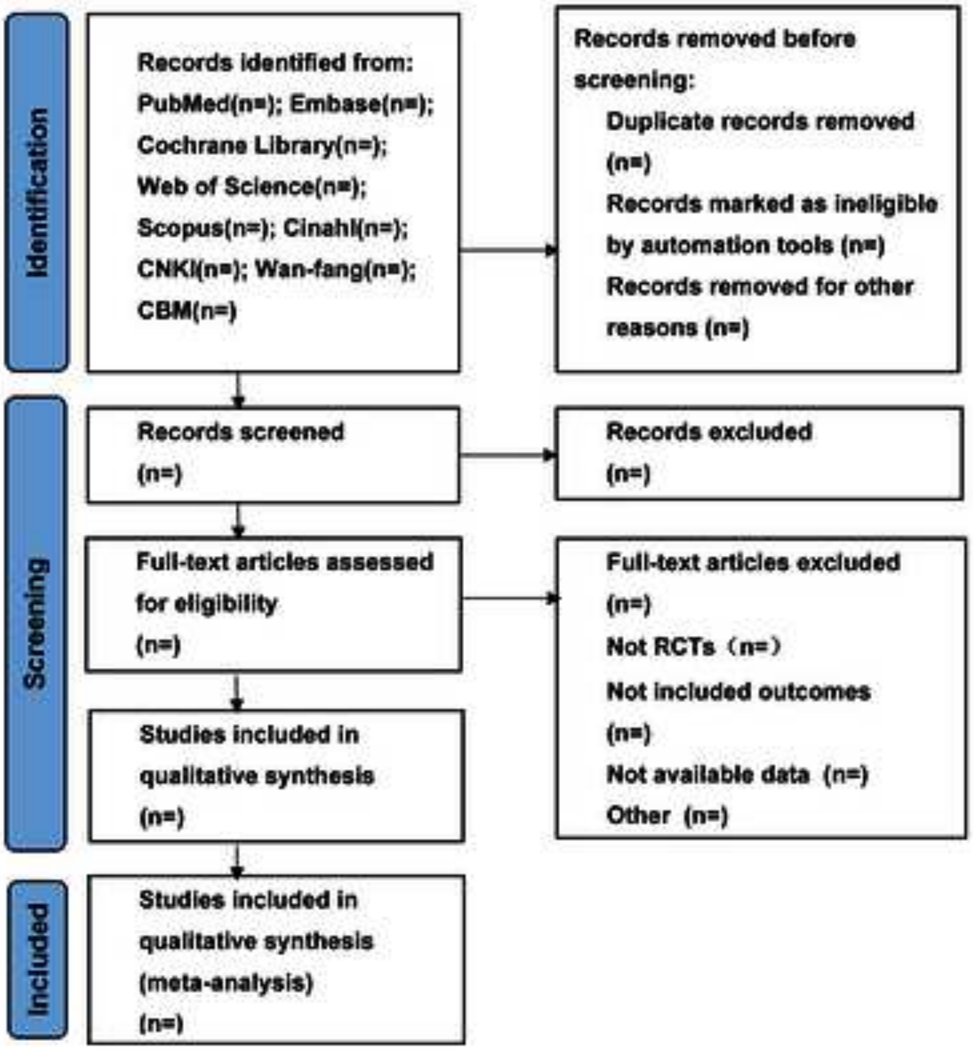

Fig.1 Flow diagram of study selection process. (n): is the number of articles that will be included at each stage.

PRISMA-P Checklist

Click here to access/download
Supporting Information
S1 Table.doc
